# Digital Health Coaching Programs Among Older Employees in Transition to Retirement: Systematic Literature Review

**DOI:** 10.2196/17809

**Published:** 2020-09-24

**Authors:** Vera Stara, Sara Santini, Johannes Kropf, Barbara D'Amen

**Affiliations:** 1 Model of Care and New Technologies IRCCS INRCA-National Institute of Health and Science on Aging Istituto di Ricovero e Cura a Carattere Scientifico Istituto Nazionale Ricovero e Cura per Anziani Ancona Italy; 2 Centre for Socio-Economic Research on Aging IRCCS INRCA-National Institute of Health and Science on Aging Istituto di Ricovero e Cura a Carattere Scientifico Istituto Nazionale Ricovero e Cura per Anziani Ancona Italy; 3 Health and Environment Austrian Institute of Technology Vienna Austria

**Keywords:** older workers, retirees, transition to retirement, healthy aging, active aging, digital coach, virtual coach, user-centered design, virtual agent, avatar, virtual personal assistant

## Abstract

**Background:**

The rapid increase of the aging population is pushing many national governments to reshape retirement legislation in order to extend older adults’ working life. Once retired, older adults can be invaluable resources for the community as family carers, as volunteers, or by returning to work. Healthy aging is one of the main conditions for being able to work longer and being active after retirement. The latter, indeed, represents a very sensitive life transition, which can entail psychological and social difficulties. Interventions for promoting older workers’ health and well-being and supporting the transition to retirement are on the top of the policy agenda of most European countries. Recently, computer-based and digital health interventions have been seen as promising means to reach this purpose.

**Objective:**

This systematic literature review aimed to explore studies on digital health coaching programs for older workers that followed a user-centered design approach and evaluated their effectiveness in providing older adults with guidance for adopting a healthy lifestyle and being active in the community.

**Methods:**

The search identified 1931 papers, and 2 relevant articles were selected by applying specific eligibility criteria.

**Results:**

To our knowledge, only few digital health coaching programs have targeted the population of older workers to date; there is an insufficient number of studies on the efficacy of such programs. The results show the difficulties of assessing the efficacy of digital coaching itself and with respect to older employees. The 2 studies suggest that digital health programs for workplaces can improve various aspects of older employees’ well-being; however, they considered health mainly from a physical perspective and neglected contextual, social, psychological, and cultural factors that can influence older workers’ health and general well-being. Future digital health coaching programs should adopt the healthy aging paradigm as a multidimensional lens for interpreting the impact of eHealth technology on aging and retirement. The literature around this issue remains at an embryonic state, and this gap needs to be filled by further investigations that apply a user-centered approach for designing the technology, test innovative research methodologies, and adopt new technical solutions for high-quality interaction design.

**Conclusions:**

Further digital health coaching programs aimed at supporting healthy and active living for older workers and retirees are necessary. The user-centered design approach is recommended in order to fully address the users’ health needs and the technological requirements throughout development. Moreover, the healthy aging perspective allows inclusion of physical, social, and psychological factors influencing the transition from work to retirement, as well as the experiences and interactions of individuals with the technology.

## Introduction

The population is aging rapidly worldwide and by 2050, or soon thereafter, the number of persons aged 65 or older will outnumber those aged 25 or under in eastern and southeastern Asia, Latin America and the Caribbean, Europe, and North America [1], thereby determining the aging of part of the workforce in most countries. In fact, between 2002 and 2018, the employment rate of persons aged 55-64, called older workers (despite the lack of general consensus on the age range among researchers and policy makers) [2], increased from 38.4% to 58.7% throughout Europe [3] and from 58.9% to 62.5% [4] in the United States.

The aging and retiring workforce can result in a loss of know-how and expertise for companies, which may negatively affect the economy and the sustainability of social security systems [5].

In order to mitigate the consequences of an aging workforce, in the last decade, many national governments of high-income countries have been changing retirement legislation to postpone the retirement age and extend older adults’ working life [6]. Continuing work in later life can indeed be considered as an aspect of *successful aging* [7], a topic on which, not surprisingly, the scientific community and the labor market has been lavishing more and more attention during the last years. An exact definition of successful aging is still a mooted point; nevertheless, it can be considered as a multidimensional research construct consisting of distinct but interrelated facets for the identification of determinants and predictors related to a favorable aging trajectory as opposed to a pathological one [8,9]. This paradigm puts emphasis on the responsibility of older people to continue to contribute to the society by working and being active [10].

Since people can be active and productive when they are in good health, it seems that a precondition of successful aging is healthy aging. The latter is not just the absence of illness, but also a “process of optimizing opportunities for physical, social and mental health to enable older people to take an active part in society without discrimination and to enjoy an independent and good quality of life [11].” Therefore, the healthy aging perspective considers every sphere of life influencing the individuals’ well-being, including social relationships, and it is particularly appropriate for looking at older employees’ experiences. Older people at the end of their working life may indeed go through a delicate phase of their existence that could be characterized by poor health but could also be characterized by psychological and relational strains at the workplace. In fact, older workers can be stigmatized in organizational settings [12] and associated with scant motivation, less alert capacity, limited productivity and flexibility [13], more resistance to change and learning [14], less reliability (from poor health), and limited digital skills [15,16]. When older workers feel stereotyped and unappreciated and when they experience effort-reward imbalance, depressive symptoms may develop [17].

In the months before retiring, the individuals’ attitudes and expectations about retirement can also be influenced by cultural patterns [18]: in countries where family culture prevails (eg, Italy), people may be more motivated to leave the labor market, because they interpret retirement as an opportunity to devote themselves to family, for example, by taking care of grandchildren or very old and non self-sufficient parents. Conversely, in other countries, where culture is more oriented to productivity as a measure of social success, such as the United States and the United Kingdom, individuals tend to remain in the labor market longer and return to work even after retirement. Moreover, some older employees plan their retirement down to the tiniest details, while others prefer not to make any plans due to fear of being unable to implement them or because they want to enjoy their freedom [18].

Once retired, older people can feel different levels of satisfaction with life, not directly associated with fulfilment of the plans and the expectations they had while working. However, satisfaction seems to be the result of the combination of multiple factors (ie, quantity and quality of interpersonal relationships and affective bonds, physical health, and financial situation within the cultural and social context) [19]. Therefore, retirement may involve both a threat of marginality and a promise of new-found freedom [20]. In the former case, the transition to retirement may be a troubling experience with anxieties, concerns, and social isolation. Conversely, in the latter case, retirement can offer scope for expanded opportunities for a new, active, and positive phase of life.

Older employees’ decision to leave work, as well as attitudes, expectations and plans for retirement, are mainly influenced by their health. In fact, the onset of chronic diseases and comorbidities are the first reasons for early retirement and are major barriers for older workers' active participation in the labor market. Indeed, according to the Survey of Health, Ageing and Retirement, in Europe, among people aged 60 to 70 years, those in good health participated in the labor market approximately twice as often as older people with 2 or more chronic diseases [21].

In light of the above, carrying out health promotion and prevention interventions targeting older workers is a prime objective of labor policy for keeping employees active and productive longer [22]. Moreover, according to Cook et al [23], there is strong evidence that well-constructed health promotion programs for employees in the workplace can be a key strategy to improve workers’ health and decrease health care costs.

One strategy to help individuals having healthier lifestyles (ie, increase physical activity, have a healthy diet, and reduce the use of tobacco and alcohol) is offering them health and lifestyle coaching through interpersonal relationship with a trainer [24]. This can motivate the individual to walk or run, eat more vegetables, limit the intake of fats, and reduce the number of cigarettes per day. Literature shows that health and lifestyle coaching programs are feasible and accepted by patients especially when based on a patient-centered approach [25]. This approach is largely adopted for older people with chronic diseases and multimorbidities [26], for prevention [27], and in primary care. Health coaching interventions are in accordance with the concept of the activation of patients, which allows them to partially determine their goals, use self-discovery or active learning processes together with content education to work toward their goals, and self-monitor behaviors to increase accountability and adherence to the program [25,28].

Since the advancement of technology has had an impressive influence on our daily living, recently coaching programs for the promotion of health have been getting increasingly more computer-based; digital health interventions are a promising approach to address older workers’ health needs [29]. Effectively, under the umbrella term of *coaching*, we usually discover studies that describe interventions delivered without a human coach but through different technologies used to drive the behavior change process. According to Sherpa Coaching Survey [30], for instance, currently only 32% of coaching is conducted face-to-face, while 68% is delivered through technological tools such as telephone (25%), webcams and Skype (25%), video conferencing in high-definition quality (10%), and e-mail coaching (8%). Despite this promising trend, few digital coaching programs have targeted the specific population of older employees by exploring their experiences with this type of technology. Consequently, studies that prove the efficacy of health programs using technology to deliver coaching services are still needed. Investigations into successful coaching systems point to the concept of user-centered design as a way of understanding the users and their needs in multiple steps of the iterative development process [31]. User-centered design is a multidisciplinary design approach based on active involvement of users to improve understanding of the consumer model. End users’ needs, capabilities, and limitations are mapped making use of a variety of methods and tools offered by this approach, and it is indeed a way to ensure that product solutions match the target’s demand, especially for health care technologies, where acceptance, usability, and reliability are essential to ensure the effectiveness of the proposed solution [32]. This mapping process is particularly important in the case of older workers since their physical and psychological characteristics are extremely specific compared to those of younger workers. Hence, any health promotion intervention directed at this age group must be matched appropriately with their particular needs and characteristics [23]. This paper, therefore, reports the findings of a systematic literature review of the studies available in the literature focusing on digital health coaching programs for older workers that were developed using the user-centered design approach.

## Methods

### Aims

This systematic literature review aimed to identify and synthesize published literature focusing on the efficacy of digital health coaching interventions specifically designed for older workers in transition to retirement (or those who just retired) and that used the user-centered design approach. To this purpose, the literature search aimed to answer the following research questions: (1) Which digital coaching interventions are effective or not effective for older employees in transition to retirement or just retired? (2) To what extent are the digital coaching interventions effective for improving the well-being of older employees in transition to retirement (or just retired)? (3) To what extent can this kind of intervention help to prevent poor health?

### Eligibility Criteria

Study inclusion criteria were (1) targeting workers aged 50 or older according to the definition of older workers [2] and expanding the latter’s age range of 5 years back to include workers in jobs such as night shifts or assembly line for greater part of the working life, and thus entitled to early retirement in most European and high-income countries [33]; (2) written in English; (3) based on user-centered design methodology to assess the impact of the digital coaching intervention through one or more of the most used methods of user-centered design reported in literature [32], such as task analysis, usability testing, field observation, interviews, questionnaires, focus groups, and randomized controlled trials. Moreover, digital coaching interventions were considered eligible if delivered by computer, smartphone or tablet, website, app or software and without the involvement of a human coach. All kinds of well being–related measures were included in this review. Articles were excluded if they (1) did not meet the eligibility criteria (ie, the population of interest and the use of technologies to deliver digital coaching); (2) did not report empirical findings.

### Search Strategy

For the literature search, the following databases were used: PubMed, Web of Science, Scopus, and IEEE. The search strategy was conducted without time limitations. Additional articles were obtained from reference lists of included studies and from Google Scholar. Databases were searched using a combination of specific terms that, in the opinion of the authors, were closely related to the topic, such as *older adult workers*, *older adults in retirement*, *transition workers in retirement*, *older employees*, *employees in retirement*, digital coach*, virtual coach*, *well-being*, *user-centered design*, *virtual agent*, *avatar*, *virtual personal assistant*. Multimedia Appendix 1 shows the search strategy in detail. The authors performed a search in each database. For each search, the above mentioned terms were combined by the use of the Boolean *AND* operator, restricting the results to articles that contained all the search terms [34,35].

### Data Collection and Extraction

According to the predefined criteria, all searches were conducted during April 2019 and May 2019 and then screened independently by the first and the last author. This screening phase was based on analysis of the title and abstract. In August 2019, full-papers of the screened publications were reviewed independently by the first and the last author. Any disagreement was resolved by including the second author in order to reach consensus for all the articles included.

### Quality Review

The Mixed Method Appraisal Tool (MMAT; version 2018), developed by Hong et al [36], was used to appraise the quality of the selected studies. In particular, MMAT provides objective tools to rigorously appraise the methodological quality of different categories of studies. The first and third authors independently appraised the methodological quality of each study; the results of each appraisal were compared and any disagreements were solved by including the second author and through discussion among the authors. A quantitative appraisal score was calculated by applying the scoring system proposed by Pluye et al [37] where the presence or absence of criteria may be scored 1 (yes) and 0 (no), respectively. Thereafter, a quality score can be calculated as a percentage: (number of yes responses divided by the number of appropriate criteria) × 100.

## Results

### General

**Figure 1:**
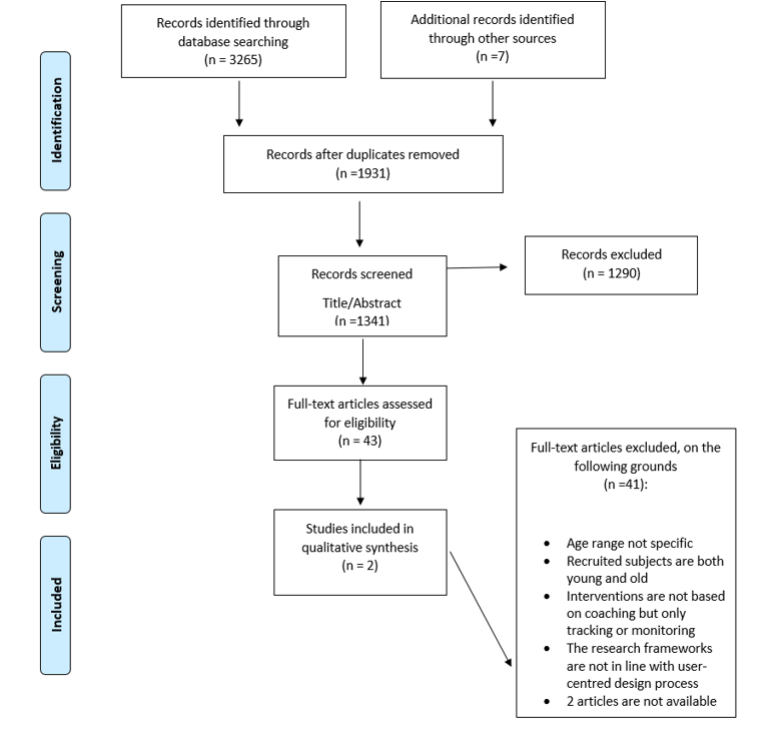
PRISMA flow diagram.

The search identified 3265 papers, and after duplicates were removed, there were 1341 articles for the initial screening. In accordance with Eden et al [38], the authors carried out the first step of the screening process based on review of titles and abstracts simultaneously. This process resulted in 277 papers. (1064 papers were excluded.) Subsequently, the authors carried out a second screening based on abstract review that resulted in 43 articles (234 papers were excluded) for the full-text selection. The abstracts were reviewed by at least 2 of the 3 researchers (VS, SS, and BD’A), and any disagreements were discussed. In cases where a resolution could not be reached, the third researcher made the final decision. This screening process based on a double review (title and abstract, abstract) allowed the authors to exclude books, books chapters, conference proceedings, or articles that did not meet the eligibility criteria. Full-text papers were downloaded for these records. From this process, the majority (n=41) were excluded because they recruited both young and old employees (n=23), the digital coaching intervention was purely a tracking or monitoring device (n=9), the empirical frameworks were not in line with user-centered design process (n=7), and 2 articles were not available; therefore, 2 papers were included [23,39]. The literature search process is shown in Figure 1.

The specifications and the main findings of the 2 studies matching the inclusion criteria, and thus included in the review, are reported and described in Table 1.

**Table 1:** Specifications and main findings of the studies included in the systematic literature review.

Reference	Purpose	Method and data collection	Sample/country	Outcomes and measures	Findings
Cook et al [23]	To evaluate the effectiveness of a web-based health program including physical activity, healthy eating, stress management, and tobacco cessation aimed specifically at older workers	Randomized controlled trial; an online survey before and 3 months after the program access	n=278 older adult employees aged 50-68, United States	1. Symptoms of distress 2. Coping with stress 3. Diet outcome expectancies 4. Barriers to a healthy diet 5. Eating practices 6. Overeating self-efficacy 7. Diet change self-efficacy 8. Planning healthy eating 9. Weight and body mass index 10. Exercise habits 11. Exercise self-efficacy 12. Self-efficacy for overcoming barriers to exercise 13. Exercise planning 14. Belief about aging 15. Tobacco use	1. Improvement of diet behavioral change self-efficacy, planning healthy eating 2. Improvement of eating practices, exercise self-efficacy, exercise planning, and aging beliefs
Irvine et al [39]	To evaluate the efficacy of a 12-week internet intervention to help sedentary older adults over 55 years of age adopt and maintain an exercise regimen	Randomized controlled trial; online survey at pretest, at 12 weeks, and at 6 months after program fruition follow-up	n=368 sedentary adults >55 years of age, United States	1. Cardiovascular activities 2. Stretching activities 3. Strengthening activities 4. Balance activities 5. Activities (minutes per week) 6. Short Form-12 physical 7. Short Form-12 mental 8. Weight and body mass index 9. Attitudes/knowledge 10. Self-efficacy 11. Behavioral attentions 12. Motivation to exercise 13. Ability to exercise 14. Barrier to exercise	1. The multivariate model indicated significant treatment effects at posttest and at 6 months 2. Improvement on 13 of 14 outcome measures 3. At 6 months, treatment participants maintained large gains compared to control on all 14 outcome measures.

### Quality Review

After calculating the score for each article [37], we synthetized methodological quality results in 3 different categories: low score, <35%; medium score, 36% to 70%; and high score, 71% to 100%.

The selected articles reached a high quality score (100%) as shown in Table 2.

**Table 2:** Quality score of the selected studies.

Reference	Type of study	Screening score	Randomized controlled clinical trial score	Total score	Appropriate criteria, n	Quantity score, %	Score category
Cook et al [23]	Randomized controlled clinical trial	2	5	7	7	100	High
Irvine et al [39]	Randomized controlled clinical trial	2	5	7	7	100	High

### Digital Program Design

The included studies were both digital interventions developed by a multidisciplinary team that focused on a significant user-centered design approach. HealthyPast50 by Cook et al [23] was a multimedia web-based program that contained information and guidance on healthy aging, diet, physical activity, stress management, and tobacco use. The program was developed specifically for adults over 50 years of age through multiple cycles of development and testing that involved older workers providing feedback on the initiative and rating the prototype content until the final realization. HealthyPast50 was based on a content management system and provided ample graphics, audio, and video contents.

Active After 55 by Irvine et al [39] was an internet-enabled CD-ROM program aimed at boosting the functional ability, mobility, and physical activity of older adults in endurance, stretching, strengthening, and balance enhancement via text and video messages. The program was developed through consultations with professionals experienced in the design and implementation of research-based exercise programs for older adults.

### Methodology for Testing and Assessing the Effectiveness of Digital Technology

The effectiveness of these studies was assessed through a randomized controlled trial and with online surveys in different phases of the assessment. The HealthyPast50 study was based on multiple outcome measures before and 3 months after the actual intervention in a sample of 278 older employees aged 50 to 68 years. The users were split randomly into a program group able to log-in to the system and a control group that did not access it. The whole sample was characterized by a high educational level and relatively high computer literacy. The program group could access the web-based program at any time during the 3-month test period at work and outside of work.

The Active After 55 study was based on 3 assessments: pretest, postintervention (12 weeks after pretest), and a 6-month follow-up, with a sample of 368 sedentary adults over 55 years of age. Authors reported that participants tended to be employed, educated, and frequent computer users with at least a middle-class income. Participants were automatically randomized into a treatment group that used the digital health program or a control group that did not have access to it. The intervention consisted in an initial 1-hour session with the Active After 55 program to obtain assistance in designing a personalized plan to follow and then 11 weekly sessions lasting at least 15 minutes in addition to weekly exercises. Both studies used an online survey to gather data in the different assessment phases.

### Main Findings of the Selected Studies

In both randomized controlled trials, the intervention group showed significant improvement in healthy behaviors compared that shown by the control group.

In the case of HealthyPast50, the working adults who used the web-based program manifested relevant enhancements over the 3-month test period on 3 out of 15 outcomes: diet behavior change, self-efficacy, and planning of healthy eating. Working adults who were given access to the web-based HealthyPast50 program showed significantly greater improvement on key health constructs over the 3-month test period compared to that shown by the individuals in the control group. In the analysis of the imputed data set, the intervention group performed significantly better than the control group on diet behavior change, self-efficacy, planning healthy eating, and mild exercises. Moreover, there were improvements in eating practices, moderate exercise, and overall exercise but these did not meet the threshold for statistical significance. As for the Active After 55 program, the hypotheses were that the intervention would be linked to changes in the exercise domains of endurance, stretching, strengthening, and balance and that it would be linked to theoretically relevant mediators of behavior change. The treatment group showed significant improvement on 13 out of 14 outcomes at posttest and on all 14 outcomes at the follow-up. The findings were consistent across an array of measures, with a large multivariate effect size at posttest and a medium multivariate effect size at the 6-month follow-up.

## Discussion

### Principal Findings

This systematic literature review was aimed at exploring the state of the art of research in the field of digital health programs sustaining the well-being of older workers in transition to retirement (or just retired) and using a user-centered design approach. Despite the wide-reaching search and review, only 2 published papers met the inclusion criteria. We provided a qualitative methodological appraisal of the 2 selected articles by means of the MMAT. Every year, a growing number of information and communication technologies emerges with the aim to provide innovative and efficient ways to help older adults in their daily life and to reduce the cost of health care. Nevertheless, there is still a paucity of studies testing the efficacy of such technologies, both for older adults in general and for older workers in transition to retirement. Hence, there is a need for more research in this field. The 2 studies included in the review strengthen the idea that multimedia web-based and internet-enabled CD-ROM programs can be effective in promoting healthy life style behaviors and in preventing poor health condition among older workers near retirement.

The findings of this systematic literature review suggest that digital health programs may help older workers to improve their health and well-being by motivating them to engage in healthy behaviors (eg, healthy diet, physical activity, and less tobacco use). Cook et al [23] and Irvine et al [39], indeed, used mainly physiological and behavioral measures for outcome assessment (eg, cardiovascular activity, body mass index, eating practices), in compliance with a concept of health as an individual physical issue. Conversely, the social, psychological, and relational [17] aspects of aging as well as the cultural patterns [18] of the participants in the studies seemed to not be monitored, and hence, were underestimated. Within the contextual factors neglected by the 2 studies, there is also the transition from work to retirement. During this period, older workers may be required to face age-related stereotypes, to upgrade their digital skills [12,17], and (as retirees) may be asked to reorganize their daily routine and their role within the family. Additionally, they are confronted with potential effects from society on health and general well-being (eg, social marginalization, anxiety, and concerns) [18,20]. In light of the above, future studies based on digital coaching programs should adopt the healthy aging paradigm [11] by adding measures for monitoring social, psychological, and cultural factors as determinants of healthy aging that are in the background of the retirement process and can influence it. This monitoring should be carried out throughout the whole study life-cycle, from the identification of the individuals’ health needs up to the development of the technology and the eHealth plan intervention. This would allow researchers to analyze the data and interpret the findings concerning users’ experience with the technology as well as with aging and retirement, from a multidimensional perspective.

A major advantage of eHealth interventions is that such interventions are easily accessible 24/7 and are usable by individuals who may not have access to traditional health promotion otherwise. When these interventions are developed around the real needs, capabilities, and limitations of older workers and tested with rigorous research methodology, the technology-based interventions demonstrate the added value of innovation in coping with healthy aging.

This idea and the review of the 2 papers inspired some suggestions for further investigation: they should use a user-centered approach for designing the technology; test innovative research methodologies; and adopt new technical solutions for high-quality interaction design.

First, digital innovation technologies can be a promising way to cope with the health and well-being deteriorations of older adults in transition from work to retirement, especially when they are designed, developed, and assessed through a user-centered approach. In fact, similar to the approach for health programs that are conducted by a person, it is preferable to adopt a patient-centered approach. Likewise, in health digital programs, it is advisable to involve patients in planning the program and designing the technology, in order to intercept their health needs and translate them into the technology requirements. This might allow patients to set health goals and program phases together with the digital coach just as they would with a human coach, thus increasing adherence to the healthy path [25]. Conversely, the marginal involvement of end users, experts, and stakeholders (who represent the articulated interdependency among individuals, organizational levels, and technological factors), along the entire process from the end-users’ needs assessment, through the identification of the technology requirements up to the realization of the product remains one of the major causes of misuse of technologies and a confounding factor in the assessment of systems [40,41]. Even though Cook et al [23] and Irvine et al [39] adopted a user-centered design approach, it seems that they limited this approach to the definition of the contents of the health program and did not use it for the development of the digital solution. In the studies [23,39], a robust method was adopted to evaluate the effectiveness of interventions using randomized controlled trials and online surveys, based on a list of standardized tests, and repeated measurements, such as pretest and posttest (before and 3 months after the end of the HealthyPast50 study and at 12 weeks and 6 months after actual program follow-up in the case of the Active After 55 intervention). Despite the significance of the randomized controlled trial approach, both studies did not provide evidence on the acceptability, usability, and learnability nor on the utility of the web-based interventions. In order to fully address these dimensions, the use of mixed methods is recommended: qualitative open-ended questions in addition to quantitative measures would be useful for capturing the perspective and the personal experiences of the users interacting with the technology.

This observation leads us to the second key point to take into consideration for future studies (ie, the use of innovative and sophisticated research methodologies). In fact, despite increasing research on the impact of technology on older people's health and well-being, most studies on this issue used small samples which neither allowed for randomized controlled trials nor generalization and transferability to other domains. Moreover, a 3-arm randomized controlled trial (ie, including one intervention group using only human coaching, a second intervention group using only digital coaching, and a control group) could offer a real framework for future research in this field. Moreover, studies comparing virtual coaching programs to web-based programs are necessary to assess which approach is more effective and which has a higher level of acceptability by older employees.

The third key point concerns the use of a variety of technical solutions to deliver digital coaching programs addressing older adults. Different workplace interventions have been developed to improve workers’ health and well-being using web-based interventions [29] and mobile apps [42]. Nevertheless, the use of embodied conversational agents, and in particular, artificial intelligence virtual coaches (ie, computer software specifically designed to work and act like a human) seems to be particularly appropriate to influence user attitudes or behaviors [43]. The advantage of this virtual coaching is almost similar to an in-person health coach offering self-management through personalized guidance and support available at any time and in any place.

The fourth consideration arising from this systematic literature review concerns the role of positive user experience, measuring people’s feelings on interacting technology in a particular context [44,45]. It is common knowledge that if the use of technology entails a negative experience for the user, as a consequence, this leads to the rejection of the system itself [46]. Therefore, it is crucial to design a high-quality interaction involving the intended users in order to identify their needs and derive the technical requirements capable of best meeting these needs [47].

### Limitations

The search was performed on 4 databases (ie Scopus, Web of Science, PubMed, and IEEE) accessed during a specific period of time (April 2019 and May 2019); in order to be more inclusive, we added references selected from Google Scholar. Given this procedure, our search could be not exhaustive and, unknowingly and unintentionally, some papers may have been omitted. Another limitation could be the keywords used for search and their combination with the Boolean *AND* operator. The selection of the terms with the Boolean operator are the results of the authors’ knowledge, which is not exhaustive. Furthermore, the narrow eligibility criteria, requiring a focus on older adults in retirement, reduced the number of studies selected.

### Comparison With Prior Work

To our best knowledge, no other reviews of digital programs are reported in the literature nor are there any on digital programs based on virtual coaching techniques targeting older workers near retirement. We could, therefore, only compare the 2 studies reviewed with literature that would be desirable for increasing knowledge in this field.

First, the reviewed studies were aimed at promoting healthy behaviors, limiting the onset of chronic diseases, and limiting the health-related costs for companies, thereby improving older worker productivity. The technologies tested by these studies were aimed at affecting the individuals’ behaviors toward physical activity, stress management, diet, and tobacco use. Future digital programs addressing older workers who are close to retirement should instead take into account not only physical health aspects related to aging itself but also psychological and social aspects that can be influenced by the transition from work to retirement. This goal could be reached by encouraging older workers to adopt strategies to counteract the side effects of retirement, such as the loss of social contacts, a sedentary lifestyle, and a decrease in intellectual stimuli. These strategies might lead older individuals onto a path of awareness of the existential change they are experiencing; to adopt a different planning of the day in order to avoid feelings of usefulness; to have attitudes more oriented to the community for the construction of bonds and friendships replacing those lost when they left work (eg, volunteering). Furthermore, in order to ensure a social dimension in the training, several individuals might share the same digital health coach. The latter might promote interpersonal relationships by helping individuals meet in person for a walk or for a social event, thereby bridging the gap between virtual and real dimensions.

### Conclusions

Alone, the recent retirement policies that have postponed retirement age cannot cope with the growing number of workers who ask to retire early due to age-related chronic diseases and multimorbidity. Moreover, workers close to retirement may need to be supported to better face this existential change.

Several studies demonstrated that technology can be very effective in promoting healthy lifestyles among older workers [23,29,39], but they often neglected the psychological and relational aspects of working in old age. Conversely, healthy aging is a multidimensional concept [11] which future research studies aiming at developing and testing digital health programs targeting older workers and retirees should refer to. A healthy aging model could be considered as a compass orienting the choice of multiple health dimensions for improvement by means of technology, including social engagement, mental health, and cultural patterns, to consider during identification of the user’ needs, technical requirements identification, and design of eHealth interventions.

The results of this systematic literature review demonstrate the difficulties of assessing the efficacy of digital coaching in itself and for older employees. While the 2 papers [23,39] suggest that workplace digital health programs show interesting results for improving various aspects of older employees’ well-being, the literature around this issue remains in an embryonic state. This gap needs to be filled by future research studies adopting a user-centered approach, innovative methodologies for assessing the technology effectiveness, new technical solutions, and high-quality interaction designs.

## Appendix

Multimedia Appendix 1 Keywords and boolean operator (AND) applied in the 4 searches used in each database.

## Abbreviations

MMAT: Mixed Methods Appraisal Tool
