# Correction: Digital Health Coaching Programs Among Older Employees in Transition to Retirement: Systematic Literature Review

**DOI:** 10.2196/25065

**Published:** 2020-12-14

**Authors:** Vera Stara, Sara Santini, Johannes Kropf, Barbara D'Amen

**Affiliations:** 1 Model of Care and New Technologies IRCCS INRCA-National Institute of Health and Science on Aging Istituto di Ricovero e Cura a Carattere Scientifico Istituto Nazionale Ricovero e Cura per Anziani Ancona Italy; 2 Centre for Socio-Economic Research on Aging IRCCS INRCA-National Institute of Health and Science on Aging Istituto di Ricovero e Cura a Carattere Scientifico Istituto Nazionale Ricovero e Cura per Anziani Ancona Italy; 3 Health and Environment Austrian Institute of Technology Vienna Austria

In “Digital Health Coaching Programs Among Older Employees in Transition to Retirement: Systematic Literature Review” (J Med Internet Res 2020;22(9):e17809) the authors noted errors in affiliations of three authors.

The affiliation of Vera Stara was originally listed as:

Model of Care and New Technologies, National Institute of Health and Science on Aging, Istituto di Ricovero e Cura a Carattere Scientifico Istituto Nazionale Ricovero e Cura per Anziani, Ancona, Italy

This has been corrected to:

Model of Care and New Technologies, IRCCS INRCA-National Institute of Health and Science on Aging, Istituto di Ricovero e Cura a Carattere Scientifico Istituto Nazionale Ricovero e Cura per Anziani, Ancona, Italy

The affiliation of Sara Santini and Barbara D'Amen was originally listed as:

Centre for Socio-Economic Research on Aging, National Institute of Health and Science on Aging, Istituto di Ricovero e Cura a Carattere Scientifico Istituto Nazionale Ricovero e Cura per Anziani, Ancona, Italy

This has been corrected to:

Centre for Socio-Economic Research on Aging, IRCCS INRCA-National Institute of Health and Science on Aging, Istituto di Ricovero e Cura a Carattere Scientifico Istituto Nazionale Ricovero e Cura per Anziani, Ancona, Italy

This change has also been made under the Corresponding Author address for Sara Santini.

The correction will appear in the online version of the paper on the JMIR Publications website on December 11, 2020, together with the publication of this correction notice. Because this was made after submission to PubMed, PubMed Central, and other full-text repositories, the corrected article has also been resubmitted to those repositories.

