# Supplementary material for: Digital Health Coaching Programs Among Older Employees in Transition to Retirement: Systematic Literature Review
Source: J Med Internet Res. 2020 Sep 24;22(9):e17809. doi: 10.2196/17809 (PMC7545329; doi:10.2196/17809)
Supplement: Multimedia Appendix 1 [file jmir_v22i9e17809_app1.docx]

## **Multimedia Appendix 1**

The combination of keywords and boolean operator (AND) applied in the four searches used in each database.

| **Searches in each database *(Scopus, PubMed, Web of Science, IEEE)*** | **Description of keywords and boolean operator** |
| --- | --- |
| Search1 | Older AND adults AND workers AND digital AND coach*  Older AND adults AND workers AND virtual AND coach*  Older AND adults AND in AND retirement AND digital AND coach*  Older AND adults AND in AND retirement AND virtual AND coach*  Transition AND workers AND in AND retirement AND digital AND coach*  Transition AND workers AND in AND retirement AND virtual AND coach*  Older AND employees AND digital AND coach*  Older AND employees AND virtual AND coach*  Employees AND in AND retirement AND digital AND coach*  Employees AND in AND retirement AND virtual AND coach* |
| Search 2 | Older AND adults AND workers AND digital AND coach* AND well-being  Older AND adults AND workers AND virtual AND coach*AND well-being  Older AND adults AND in AND retirement AND digital AND coach* AND well-being  Older AND adults AND in AND retirement AND virtual AND coach*AND well-being  Transition AND workers AND in AND retirement AND digital AND coach* AND well-being  Transition AND workers AND in AND retirement AND virtual AND coach*AND well-being  Older AND employees AND digital AND coach* AND well-being  Older AND employees AND virtual AND coach*AND well-being  Employees AND in AND retirement AND digital AND coach*AND well-being  Employees AND in AND retirement AND virtual AND coach*AND well-being |
| Search 3 | Older AND adults AND workers AND digital AND coach* AND user-centered AND design  Older AND adults AND workers AND virtual AND coach*AND user-centered AND design  Older AND adults AND in AND retirement AND digital AND coach* AND user-centered AND design  Older AND adults AND in AND retirement AND virtual AND coach*AND user-centered AND design  Transition AND workers AND in AND retirement AND digital AND coach* AND user-centered AND design  Transition AND workers AND in AND retirement AND virtual AND coach*AND user-centered AND design  Older AND employees AND digital AND coach* AND user-centered AND design  Older AND employees AND virtual AND coach*AND user-centered AND design  Employees AND in AND retirement AND digital AND coach*AND user-centered AND design  Employees AND in AND retirement AND virtual AND coach*AND user-centered AND design |
| Search 4 | Older AND adults AND workers AND virtual AND agent AND user-centered AND design  Older AND adults AND workers AND avatar AND user-centered AND design  Older AND adults AND workers AND virtual AND personal AND assistant AND user-centered AND design  Older AND adults AND in AND retirement AND virtual AND agent AND user-centered AND design  Older AND adults AND in AND retirement AND avatar AND user-centered AND design  Older AND adults AND in AND retirement AND virtual AND personal AND assistant AND user-centered AND design  Transition AND workers AND in AND retirement AND virtual AND agent AND user-centered AND design  Transition AND workers AND in AND retirement AND avatar AND user-centered AND design  Transition AND workers AND in AND retirement AND virtual AND personal AND assistant AND user-centered AND design  Older AND employees AND virtual AND agent AND user-centered AND design  Older AND employees AND avatar AND user-centered AND design  Older AND employees AND virtual AND personal AND assistant AND user-centered AND design  Employees AND in AND retirement AND virtual AND agent AND user-centered AND design  Employees AND in AND retirement AND avatar AND user-centered AND design  Employees AND in AND retirement AND virtual AND personal AND assistant AND user-centered AND design |
